# Supplementary material for: Dynamics of cooperation in concurrent games
Source: Nat Commun. 2025 Feb 11;16:1524. doi: 10.1038/s41467-025-56083-7 (PMC11814277; doi:10.1038/s41467-025-56083-7)
Supplement: Supplementary file 1 — Supplementary Information [file 41467_2025_56083_MOESM1_ESM.pdf]

Supplementary Information

Dynamics of cooperation in concurrent games

Charlotte S. L. Rossetti[1], Oliver P. Hauser[2,†], Christian Hilbe[1,†]

[1]Max Planck Research Group on the Dynamics of Social Behavior,
Max Planck Institute for Evolutionary Biology, 24306 Plön, Germany

[2]Department of Economics, University of Exeter, Exeter, EX4 4PU, UK

[†] These authors contributed equally.

Contents

1 Supplementary literature **2**
- 1.1 Theoretical work . . . 2
- 1.2 Experimental work . . . 4

2 Supplementary methods **5**
- 2.1 Baseline model . . . 5
  - 2.1.1 Control treatment . . . 5
  - 2.1.2 Same-partner treatment . . . 7
  - 2.1.3 Different-partners treatment . . . 8
  - 2.1.4 Trembling-hand errors . . . 9
- 2.2 Modelling cognitive constraints and alternative behavioral processes . . . 10
  - 2.2.1 Imperfect recall . . . 10
  - 2.2.2 Behavioral spillovers . . . 12
  - 2.2.3 Preferential updating . . . 13
  - 2.2.4 Updating under narrow bracketing . . . 14
- 2.3 Experimental methods . . . 15
  - 2.3.1 Experimental procedures . . . 15
  - 2.3.2 Statistical methods . . . 16
  - 2.3.3 Additional results . . . 17

3 Supplementary Figures **18**

4 Supplementary References **33**

1 Supplementary literature

To a large extent, evolutionary game theory takes a reductionist approach. It aims to understand social behavior by exploring the dynamics of individual games. This is also the approach of most text books (1–4) and reviews (5–10). Instead, here we are interested in describing how people act if they are involved in several games concurrently (possibly with different interaction partners). In the following, we summarize the previous theoretical and experimental literature on this topic, and we describe how our approach differs from this literature.

1.1 Theoretical work

We study direct reciprocity in concurrently ongoing repeated games. This work is naturally related to several strands of the evolutionary game theory literature.

Evolutionary dynamics of multi-games. Several researchers have explored the evolutionary dynamics in concurrent non-repeated games (11–14). Here, players engage in two or more one-shot games concurrently. This literature asks to which extent the dynamics and the equilibria of such 'multi-games' can be directly inferred from the constituent games. While the equilibria of multi-games are typically directly related to the equilibria of the individual one-shot games (11), the overall evolutionary dynamics may differ. In particular, multi-games may lead to evolutionary oscillations that have no analogue in any of the constituent one-shot games (12). While this work provides important insights into the evolutionary dynamics of one-shot games, it does not extend to reciprocal interactions. In these models, individuals have no possibility (or interest) to strategically link their behavior across different games. Similarly, these models do not study the effect of spillovers from one game to another.

Social dilemmas with punishment or rewards. There is also abundant work in which a social dilemma is coupled with a subsequent game in which individuals can reward or punish each other (15–21). Such social dilemmas with incentives share some similarities with our same-partner treatment, to the extent that individuals can use one game (the rewarding or the punishment game) to incentivize additional cooperation in the other. A crucial difference, however, is that the second punishment/reward stage is explicitly designed to strategically influence co-players to cooperate. In contrast, we are interested in how people approach concurrently ongoing games when there is no exogenous indication that behavior in one game might or even should be tied to the behavior in the other.

Direct reciprocity. While there is a vast literature on the evolution of reciprocal cooperation, most studies assume that individuals only interact in one repeated game at a time, or that they treat all their games as independent (22–40). The setup of these studies thus corresponds to the setup of our control treatment. In addition, there is literature on so-called stochastic games (41–43). This literature asks how people

cooperate when their behavior in the current round has an influence on the game played in the next round. For example, depending on the players' previous actions, players might either play a high-benefit or a low-benefit game. Importantly, however, players only engage in a single game in every round. Compared to these studies, we ask: does the presence of other ongoing games enhance or suppress cooperation? How can we model the different ways in which behavior in one game may consciously or subconsciously affect behavior in another game?

Some of these questions have been taken up by the industrial economics literature, when asking how firms compete in several distinct markets (44, 45). These studies conceptualize the competition across different markets as formally independent repeated games, similar to our same-partner treatment. Assuming that markets and firms are symmetric, this literature finds an irrelevance result (44): multi-market contact does not allow firms to collude more effectively, compared to the possibilities given by each isolated game. The respective papers, however, take an equilibrium perspective. They characterize optimal behavior among rational players. In contrast, we are interested in how people learn to play strategically over time (far from equilibrium). To this end, we also formulate learning processes that aim to capture some of the cognitive constraints and biases people may be susceptible to.

Two studies from the evolutionary game theory literature are most closely related to what we do. First, Reiter et al (46) consider players arranged on a network, who interact in a repeated prisoner's dilemma with each of their neighbors. The players' strategies treat each game as independent (as in our control treatment). That is, the strategies only depend on the previous action of the respective neighbor. However, the model allows for 'crosstalk' between different games. When a crosstalk event occurs, a player who intends to defect against one neighbor may mistakenly also defect against another neighbor (similarly for cooperation). In line with the results of our model extension on exogenous spillovers, the study finds that higher crosstalk rates undermine the evolution of cooperation. Importantly, however, their model does not study the endogenous evolution of linkage between independent games. Instead crosstalk between games is included by an exogenous parameter.

Second, Donahue et al (47) describe the equilibria of our same-partner treatment. As in our case, there are two players who engage in two repeated games in parallel. Players can condition their behavior in either game on the previous outcome of both games. As in our simulation of the baseline scenario (**Fig. 2**), they find that concurrent games can lead to strategic linkage. This linkage in turn can promote cooperation compared to the control treatment. Our model generalizes this work substantially: (*i*) We introduce a consistent framework that allows us to compare strategic effects of linking different games with non-strategic effects (by comparing the same-partner and the different-partners treatment). (*ii*) We explore the impact of several plausible biases and psychological constraints (see also Section 2.2). (*iii*) By designing and analyzing a behavioral experiment based on our model, we compare the theoretical predictions to empirical evidence.

1.2 Experimental work

Direct reciprocity. Just as in the theoretical papers mentioned above, most experimental studies look at individual games in isolation (48). One notable exception is spatial games played on a network where players interact with their neighbors (49–51). In these spatial prisoner's dilemma, players have been found to reciprocate cooperation based on the number of their neighbours who cooperated on the previous round and their own previous action (52). Nonetheless, as the number of co-players increases, cooperation decays (53–55). However, in these experiments players' actions are the same for all neighbors, meaning they cannot tailor their choice of action to each individual neighbor. Further experiments on dynamic networks find that players are eager to cut ties to non-cooperative neighbors when they are offered the possibility (56, 57), even when doing so is costly (58).

Spillover in multi-games. Nonetheless, there is a small literature on on simultaneous games outside of a network, specifically looking at strategic spillovers between two games. In the economics literature, a range of papers tests whether strategies from two distinct games might influence one another when playing concurrently, by comparing two different coordination games (59), a Public Goods Game and a lottery contest (60), and different payoff matrices of non-cooperative games (61, 62). Another group of studies look at spillovers in cooperative behaviour specifically, by comparing Prisoner's dilemma games differing in payoffs (63) or Public goods games with varying incentives (64–66). However, they focus on behavioral spillovers between games, but not reciprocity and strategic linkage in particular.

Angelovski *et al* (67) consider a setup similar to our different-partners treatment. They investigate how people interact in two simultaneous 2-players public goods games. Players are arranged on a circle, meaning that each player interacts with two neighbors. The two games differ in their incentives: one has a high incentive, the other a low one. In addition, they include three controls where (1) both games have the high incentive, (2) both games have the low incentive, and (3) both games have the average of the two incentives. The results show that cooperation in the asymmetric public goods game matches the level found in the control with high incentives in both games, meaning that the game with the higher incentive is able to lift cooperation in the game with lower incentive. They also check whether contributions in one game depend on how much the neighbor in the other game contributed on average. Average contributions to each neighbor correlate with each other and are behaviourally interdependent.

Finally, one paper contemporaneous to ours, on the repeated prisoner's dilemma, uses a similar design to us. Laferrière *et al* (68) look at cooperation in repeated games with different payoffs played simultaneously with either the same or two different co-players. The games only differ in their temptation and sucker payoff while the reward and punishment payoffs stay the same. The paper is focused on experiments and has no evolutionary game theory component. In line with our results, they find that average cooperation is the same whether the interactions are with the same or different co-players. However, they do not include a single game control to understand the effect of playing two games simultaneously. Our experiment includes such a control where players interact in a standard repeated prisoner's dilemma with either a high or low

benefit of cooperation.

2 Supplementary methods

2.1 Baseline model

We consider two stage games, the high-benefit game (*high game*, $\mathtt{H}$) and the low-benefit game (*low game*, $\mathtt{L}$). In either game, players can either cooperate (C) or defect (D). A cooperator pays a cost $c > 0$ in order for the co-player to get a benefit $b_k > c$. This benefit b_k depends on the type of game, $k \in \{\mathtt{H}, \mathtt{L}\}$. As implied by their names, the high game has a larger benefit $b_{\mathtt{H}} \geq b_{\mathtt{L}}$. Overall, each stage game has the payoff matrix

$$\begin{array}{c} \\ C \\ D \end{array} \begin{array}{c} \begin{array}{cc} C & D \end{array} \\ \begin{pmatrix} b_k - c & -c \\ b_k & 0 \end{pmatrix} \end{array} \qquad [1]$$

In particular, both games have the characteristics of a prisoner's dilemma. Mutual cooperation yields the highest total payoff for the two players, but for each individual player, cooperation is dominated. Players engage in these donation games for infinitely many rounds. We refer to each individual iterated game as a *repeated game*. Overall, we analyze three treatments that differ in two aspects:

1. Players either engage in one repeated game, or in both repeated games.
2. When they engage in both games, they either face the same co-player in both games, or a different co-player in each game.

In all cases, players use reactive strategies to make their decisions (69, 70). This means a player's action in any game only depends on the co-players' actions in the previous round (independent of all earlier actions). When players engage in both games, we refer to the resulting supergame as a *concurrent game*. Here players need to make two decisions each round, one for each stage game. Decisions that happen in one repeated game may influence decisions in the other. In the following, we introduce the three treatments in detail and we explain how to compute the players' payoffs in each case.

2.1.1 Control treatment

Setup and strategies. In the control treatment, players only engage in one repeated game at a time. This is the case that is usually considered in the literature. To be consistent with the other treatments, we assume the control treatment involves four players. Players 1 and 2 interact in the repeated game with high benefits; players 3 and 4 engage in the low-benefit game. For either repeated game $k \in \{\mathtt{H}, \mathtt{L}\}$, strategies take the form $\mathbf{p^k} = (p^k_C, p^k_D) \in [0, 1]^2$. Here, p^k_a refers to the player's probability to cooperate depending on the

co-player's previous action $a \in \{C, D\}$. For example, $\mathbf{p^k} = (0,0)$ refers to the strategy ALLD. As another example, $\mathbf{p^k} = (1,0)$ implements the strategy TFT (Tit-for-Tat).

While we assume for the control treatment that each player only engages in one game at a time, we obtain equivalent results if they play both games (either with the same partner or with different partners), provided players can be assumed to treat both games as independent (see **Fig. S1**). That is, we obtain an equivalent formulation of our control treatment if we assume players engage in both games simultaneously, but strategies are restricted to take the form $\mathbf{p} = (p_C^{\mathrm{H}}, p_D^{\mathrm{H}};\ p_C^{\mathrm{L}}, p_D^{\mathrm{L}})$. Here, p_a^k is the player's cooperation probability in game k, depending on the co-player's previous action in the respective game only. The theoretical results of this alternative formulation might be somewhat easier to compare to the other treatments (because then all treatments would involve two repeated games for each player). However, this alternative formulation is more difficult to implement experimentally. Therefore, we use the first formulation throughout, as depicted in our **Fig. 1b**.

Calculation of payoffs. We describe how to compute payoffs for players 1 and 2. Payoffs for the other two players are computed equivalently. Suppose player 1 uses strategy $\mathbf{p^1} = (p_C^1, p_D^1)$, whereas player 2 uses strategy $\mathbf{p^2} = (p_C^2, p_D^2)$. To compute payoffs, we represent the repeated game as a Markov chain (see, for example, Ref. 3). The possible states of the Markov chain are the possible outcomes of a single stage game, (C,C), (C,D), (D,C), (D,D). Here, the first and the second letter refer to the actions of player 1 and player 2, respectively. Given the players' strategies, the transition matrix takes the following form (3),

$$M(\mathbf{p^1}, \mathbf{p^2}) = \begin{pmatrix} p_C^1\, p_C^2 & p_C^1\,(1-p_C^2) & (1-p_C^1)\, p_C^2 & (1-p_C^1)\,(1-p_C^2) \\ p_C^1\, p_D^2 & p_C^1\,(1-p_D^2) & (1-p_C^1)\, p_D^2 & (1-p_C^1)\,(1-p_D^2) \\ p_D^1\, p_C^2 & p_D^1\,(1-p_C^2) & (1-p_D^1)\, p_C^2 & (1-p_D^1)\,(1-p_C^2) \\ p_D^1\, p_D^2 & p_D^1\,(1-p_D^2) & (1-p_D^1)\, p_D^2 & (1-p_D^1)\,(1-p_D^2) \end{pmatrix}. \qquad [2]$$

As the two players interact in this game for many rounds, play converges to an invariant distribution $\mathbf{v} = (v_{CC}, v_{CD}, v_{DC}, v_{DD})$. Each entry v_{a^1,a^2} gives the long-run probability to observe rounds in which the first player chooses a^1 whereas the second player chooses a^2. For $\mathbf{p^1}, \mathbf{p^2} \in (0,1)^2$, this invariant distribution is unique. By the theorem of Perron-Frobenius, it is given by the solution of the eigenvector problem

$$\mathbf{v} = \mathbf{v} M(\mathbf{p^1}, \mathbf{p^2}). \qquad [3]$$

Based on this invariant distribution, we can compute the players' average cooperation rates as

$$\gamma^1 = v_{CC} + v_{CD} \quad \text{and} \quad \gamma^2 = v_{CC} + v_{DC}. \qquad [4]$$

In the control treatment, these cooperation rates can be computed explicitly (3), as a function of the players'

reactive strategies $\mathbf{p^{1k}}$ and $\mathbf{p^{2k}}$,

$$\gamma^1 = \frac{p_D^1 + (p_C^1 - p_D^1)\cdot p_D^2}{1-(p_C^1-p_D^1)(p_C^2-p_D^2)} \quad \text{and} \quad \gamma^2 = \frac{p_D^2 + (p_C^2 - p_D^2)\cdot p_D^1}{1-(p_C^1-p_D^1)(p_C^2-p_D^2)}. \tag{5}$$

As a result, we can compute the players' average payoffs for the repeated game as

$$\pi^1 = b_{\mathrm{H}}\cdot\gamma^2 - c\cdot\gamma^1 \quad \text{and} \quad \pi^2 = b_{\mathrm{H}}\cdot\gamma^1 - c\cdot\gamma^2. \tag{6}$$

2.1.2 Same-partner treatment

Setup and strategies. Here, again we consider two pairs of players, players 1 and 2, and players 3 and 4. However, this time each pair engages in both repeated games in parallel. It follows that one player's action in one game may affect the co-player's next action in the other game. Such a situation has been previously referred to as a 'multichannel game' (47). A player's strategy for repeated game k is a 4-tuple $\mathbf{p^k} = (p^k_{CC}, p^k_{CD}, p^k_{DC}, p^k_{DD}) \in [0,1]^4$. An entry $p^k_{a^{\mathrm{H}},a^{\mathrm{L}}}$ refers to the player's cooperation probability in game k, depending on the co-player's previous actions in both the high and the low game. We note that this strategy space contains the strategy space of the control treatment as a special case. For example, a strategy $\mathbf{p}^{\mathrm{H}} = (p^{\mathrm{H}}_C, p^{\mathrm{H}}_D)$ of the control treatment can be represented as $\mathbf{p}^{\mathrm{H}} = (p^{\mathrm{H}}_C, p^{\mathrm{H}}_C, p^{\mathrm{H}}_D, p^{\mathrm{H}}_D)$ within the same-partner treatment. This reflects a case in which a player could, in principle, react to the co-player's previous actions in both games but decides to only react to the actions in the respective game. A strategy for the concurrent game is a pair $\mathbf{p} = (\mathbf{p}^{\mathrm{H}}, \mathbf{p}^{\mathrm{L}}) \in [0,1]^8$ that tells the player what to do in each repeated game.

Calculation of payoffs. As in the first treatment, we compute payoffs with a Markov chain approach. Without loss of generality, consider the first two players, with strategies $\mathbf{p^{1k}}$ and $\mathbf{p^{2k}}$, respectively. Because players no longer treat each game as independent, a state is now a 4-tuple $\mathbf{a} = (a^{1\mathrm{H}}, a^{2\mathrm{H}}, a^{1\mathrm{L}}, a^{2\mathrm{L}})$ with $a^{ik} \in \{C, D\}$. Each state corresponds to a possible outcomes of a single round, with the entries describing the players' actions in each game. For example (C, C, D, C) refers to a state in which both players cooperated in the high game, but only player 2 cooperated in the low game. There are $2^4 = 16$ states in total. For two possible states $\mathbf{a} = (a^{1\mathrm{H}}, a^{2\mathrm{H}}, a^{1\mathrm{L}}, a^{2\mathrm{L}})$ and $\tilde{\mathbf{a}} = (\tilde{a}^{1\mathrm{H}}, \tilde{a}^{2\mathrm{H}}, \tilde{a}^{1\mathrm{L}}, \tilde{a}^{2\mathrm{L}})$, we can compute the transition probability that players move from state $\mathbf{a}$ to $\tilde{\mathbf{a}}$ within one round. The respective transition probability is a product of four factors,

$$m_{\mathbf{a},\tilde{\mathbf{a}}} = y^{1\mathrm{H}}_{a^{2\mathrm{H}}a^{2\mathrm{L}}} \cdot y^{2\mathrm{H}}_{a^{1\mathrm{H}}a^{1\mathrm{L}}} \cdot y^{1\mathrm{L}}_{a^{2\mathrm{H}}a^{2\mathrm{L}}} \cdot y^{2\mathrm{L}}_{a^{1\mathrm{H}}a^{1\mathrm{L}}}. \tag{7}$$

These four factors correspond to the decisions that the two players make in each of the two games. The first factor is the probability that player 1 chooses the action required by $\tilde{\mathbf{a}}$ in the high game,

$$y^{1\mathrm{H}}_{a^{2\mathrm{H}}a^{2\mathrm{L}}} := \begin{cases} p^{1\mathrm{H}}_{a^{2\mathrm{H}}a^{2\mathrm{L}}} & \text{if } \tilde{a}^{1\mathrm{H}} = C \\ 1 - p^{1\mathrm{H}}_{a^{2\mathrm{H}}a^{2\mathrm{L}}} & \text{if } \tilde{a}^{1\mathrm{H}} = D. \end{cases} \tag{8}$$

The three other factors in Eq. [7] are defined analogously. Again, we collect these transition probabilities in a 16×16 matrix $M(\mathbf{p^1}, \mathbf{p^2}) = (m_{\mathbf{a},\tilde{\mathbf{a}}})$. By computing the invariant distribution $\mathbf{v} = (v_{\mathbf{a}})$ of this matrix, we infer how often the two players visit each state $\mathbf{a} = (a^{1\mathtt{H}}, a^{2\mathtt{H}}, a^{1\mathtt{L}}, a^{2\mathtt{L}}) \in \{C, D\}^4$ over the course of the game. Based on this invariant distribution, we compute the average cooperation rate of player i in game k. To this end, we sum up over all possible states in which player i cooperates in game k,

$$\gamma^{ik} = \sum_{\mathbf{a} \in \{C,D\}^4} v_{\mathbf{a}} \cdot 1_{\{a^{ik}=C\}}. \qquad [9]$$

Here, 1_P is an indicator function. Its value is one if the statement P is true, and it is zero otherwise. Based on these cooperation rates we define the player's payoffs in repeated game $k \in \{\mathtt{H}, \mathtt{L}$ as

$$\pi^{1k} = b_k \cdot \gamma^{2k} - c \cdot \gamma^{1k} \quad \text{and} \quad \pi^{2k} = b_k \cdot \gamma^{1k} - c \cdot \gamma^{2k}. \qquad [10]$$

We define the payoffs for the concurrent game by adding up the payoffs for each repeated game,

$$\pi^1 = \pi^{1\mathtt{H}} + \pi^{1\mathtt{L}} \quad \text{and} \quad \pi^2 = \pi^{2\mathtt{H}} + \pi^{2\mathtt{L}}. \qquad [11]$$

The payoffs for players 3 and 4 can be computed with the same algorithm.

2.1.3 Different-partners treatment

Setup and strategies. In this treatment, players have different interaction partners for the high and the low game. Specifically, we consider four players, with interactions as displayed in **Fig. 1** of the main text. For example, player 1's interaction partner in the high game is player 2, whereas the interaction partner in the low game is player 3. Similar to the same-partner treatment, a player's action in one game may depend on the previous outcome of the other game. That is, a strategy for game k again takes the form $\mathbf{p^k} = (p^k_{CC}, p^k_{CD}, p^k_{DC}, p^k_{DD}) \in [0,1]^4$. Each entry $p^k_{a^{\mathtt{H}},a^{\mathtt{L}}}$ indicates the player's probability to cooperate in game k, depending on the previous action $a^{\mathtt{H}}$ of the co-player in the high game, and on the action $a^{\mathtt{L}}$ of the co-player in the low game. Again, a strategy for the concurrent game is a pair $\mathbf{p} = (\mathbf{p}^{\mathtt{H}}, \mathbf{p}^{\mathtt{L}}) \in [0,1]^8$. It tells the player what to do in each of the two games.

Calculation of payoffs. As before, payoffs are computed with a Markov chain approach. However, because we no longer have two distinct pairs of players, the state space is yet again bigger. This time, the set of states consist of all 8-tuples $\mathbf{a} = (a^{1\mathtt{H}}, a^{2\mathtt{H}}, a^{3\mathtt{H}}, a^{4\mathtt{H}}, a^{1\mathtt{L}}, a^{2\mathtt{L}}, a^{3\mathtt{L}}, a^{4\mathtt{L}}) \in \{C, D\}^8$. For example, a tuple $\mathbf{a}$ with $a^{3\mathtt{L}} = C$ refers to a round in which player 3 cooperates in the low game with the respective interaction partner (player 1). Because there are now eight (independent) decisions made each round, the transition probability from state $\mathbf{a}$ to $\tilde{\mathbf{a}}$ is a product of eight factors,

$$m_{\mathbf{a},\tilde{\mathbf{a}}} = y^{1\mathtt{H}}_{a^{2\mathtt{H}}a^{3\mathtt{L}}} \cdot y^{2\mathtt{H}}_{a^{1\mathtt{H}}a^{4\mathtt{L}}} \cdot y^{3\mathtt{H}}_{a^{4\mathtt{H}}a^{1\mathtt{L}}} \cdot y^{4\mathtt{H}}_{a^{3\mathtt{H}}a^{2\mathtt{L}}} \cdot y^{1\mathtt{L}}_{a^{2\mathtt{H}}a^{3\mathtt{L}}} \cdot y^{2\mathtt{L}}_{a^{1\mathtt{H}}a^{4\mathtt{L}}} \cdot y^{3\mathtt{L}}_{a^{4\mathtt{H}}a^{1\mathtt{L}}} \cdot y^{4\mathtt{L}}_{a^{3\mathtt{H}}a^{2\mathtt{L}}}. \qquad [12]$$

The entries $y^{ik}_{a^{\mathrm{H}}a^{\mathrm{L}}}$ are defined the same way as before, see Eq. [8]. The respective transition matrix $M(\mathbf{p^1}, \mathbf{p^2}, \mathbf{p^3}, \mathbf{p^4}) = (m_{\mathbf{a},\tilde{\mathbf{a}}})$ that summarizes all these transition probabilities is now of size 256×256 (in particular, the players' payoffs are now more computationally expensive to derive). However, the remaining steps are analogous to the previous treatments. Again, we first compute the players' average cooperation rates in each game by summing up over all relevant states $\mathbf{a} = (a^{1\mathrm{H}}, a^{2\mathrm{H}}, a^{3\mathrm{H}}, a^{4\mathrm{H}}, a^{1\mathrm{L}}, a^{2\mathrm{L}}, a^{3\mathrm{L}}, a^{4\mathrm{L}})$,

$$\gamma^{ik} = \sum_{\mathbf{a} \in \{C,D\}^8} v_{\mathbf{a}} \cdot 1_{\{a^{ik}=C\}}. \qquad [13]$$

Based on these eight cooperation rates, we can compute the players' payoffs in each game as follows,

$$\begin{aligned}
\pi^{1\mathrm{H}} &= b_{\mathrm{H}} \cdot \gamma^{2\mathrm{H}} - c \cdot \gamma^{1\mathrm{H}}, & \pi^{1\mathrm{L}} &= b_{\mathrm{L}} \cdot \gamma^{3\mathrm{L}} - c \cdot \gamma^{1\mathrm{L}},\\
\pi^{2\mathrm{H}} &= b_{\mathrm{H}} \cdot \gamma^{1\mathrm{H}} - c \cdot \gamma^{2\mathrm{H}}, & \pi^{2\mathrm{L}} &= b_{\mathrm{L}} \cdot \gamma^{4\mathrm{L}} - c \cdot \gamma^{2\mathrm{L}},\\
\pi^{3\mathrm{H}} &= b_{\mathrm{H}} \cdot \gamma^{4\mathrm{H}} - c \cdot \gamma^{3\mathrm{H}}, & \pi^{3\mathrm{L}} &= b_{\mathrm{L}} \cdot \gamma^{1\mathrm{L}} - c \cdot \gamma^{3\mathrm{L}},\\
\pi^{4\mathrm{H}} &= b_{\mathrm{H}} \cdot \gamma^{3\mathrm{H}} - c \cdot \gamma^{4\mathrm{H}}, & \pi^{4\mathrm{L}} &= b_{\mathrm{L}} \cdot \gamma^{2\mathrm{L}} - c \cdot \gamma^{4\mathrm{L}}.
\end{aligned} \qquad [14]$$

The overall payoff of player i in the concurrent game is the sum $\pi^i = \pi^{i\mathrm{H}} + \pi^{i\mathrm{L}}$.

2.1.4 Trembling-hand errors

The strategies introduced above describe which actions the players *wish* to take. However, actions may be subject to errors. For example, players may intend to cooperate, but they may fail because of a lack of attention. Similarly, players may misimplement their intended action because of a *trembling hand* (71). The assumption of trembling-hand errors is fairly common in the evolutionary literature on repeated games (9). In addition to making the model more realistic, errors have two useful mathematical implications. First, errors ensure that all finite histories of a repeated game are visited with a positive probability. As a consequence, each entry of a player's strategy has an effect on the player's payoff (there are no entries that are neutral merely because the respective history is never visited). Second, errors ensure that the Markov chains described in the previous sections are ergodic, irrespective of the players' strategies. This implies that payoffs are well-defined even when the unperturbed Markov chain has multiple absorbing states.

We assume trembling-hand errors occur with a constant probability $\varepsilon_{\mathrm{TH}}$, and they affect cooperative and defective actions alike. As a result, a player's *nominal strategy* $\mathbf{p^k}$ for game k translates into an *effective strategy* $\mathbf{p^k_\varepsilon}$, with

$$\mathbf{p^k_\varepsilon} := (1 - \varepsilon_{\mathrm{TH}})\mathbf{p^k} + \varepsilon_{\mathrm{TH}}(\mathbf{1} - \mathbf{p^k}). \qquad [15]$$

Here, $\mathbf{1}$ is a vector that has the same size as $\mathbf{p^k}$, but with all entries being equal to one. As an example, under this transformation, the repeated-game strategy Tit-for-Tat (1,0) in the control treatment is mapped to the effective strategy $(1 - \varepsilon_{\mathrm{TH}}, \varepsilon_{\mathrm{TH}})$. More generally, this transformation takes a strategy $\mathbf{p^k} \in [0,1]^n$,

and maps it into the interior of this strategy space, $\mathbf{p}^{\mathbf{k}}_{\varepsilon} \in [\varepsilon_{\text{TH}}, 1-\varepsilon_{\text{TH}}]^n$. The above strategy transformation works for all three considered treatments. We can compute the players' (effective) payoffs by simply taking the effective strategies (instead of the nominal strategies) as the input in the respective payoff algorithms.

2.2 Modelling cognitive constraints and alternative behavioral processes

Once players interact in several games in parallel, their behavior may be subject to cognitive constraints that cannot be studied within the classical framework of (independently) repeated games. In the following, we consider some of these constraints, and we discuss how they can be incorporated into our framework for the same-partner treatment and the different-partners treatment, respectively.

2.2.1 Imperfect recall

Motivation. When engaging in several interactions in parallel, people may confuse the outcome of one interaction with the outcome of another. This effect is best documented in studies where participants need to memorize the outcome of different social dilemmas with changing interaction partners (72, 73). As one may expect, these studies find that it is more difficult to correctly recollect one interaction partner's past decision when several other interactions (with different outcomes) occurred in the meanwhile.

Imperfect recall can undermine reciprocal cooperation because it restricts the players' ability to give targeted responses. This may also affect the predictions of our baseline model. As an example, consider a player whose co-player in the high game defected in the previous round, and whose co-player in the low game cooperated. With perfect recall, such a player would react by cooperating with probability $p^{\mathtt{H}}_{DC}$ and $p^{\mathtt{L}}_{DC}$, respectively. With imperfect recall, this player may confuse the outcomes of the last round, and choose to cooperate with probability $p^{\mathtt{H}}_{CD}$ and $p^{\mathtt{L}}_{CD}$ instead. The impact of this cognitive constraint depends on the exact values of the cooperation probabilities. The impact is small when $p^{k}_{CD} \approx p^{k}_{DC}$ for both $k \in \{\mathtt{H}, \mathtt{L}\}$. It can be substantial when the cooperation probabilities differ considerably.

Incorporating this kind of imperfect recall into our model is not as straightforward as incorporating trembling-hand errors in Section 2.1.4. There we assumed that trembling-hand errors affect each game of a player independently. An error in one game does not increase or decrease the likelihood that a similar error occurs in the other game. As a result, we can model the effect of errors by simply replacing nominal strategies by effective strategies, see Eq. [15]. In contrast, imperfect recall affects both games simultaneously. In the following, we describe how confusion can be integrated into our framework, both for the same-partner and the different-partners treatment.

Same-partner treatment. We suppose that in any given round, each player may be subject to imperfect recall with probability $\varepsilon_{\text{IR}} \geq 0$. Let $\mathbf{a} = (a^{1\mathtt{H}}, a^{2\mathtt{H}}, a^{1\mathtt{L}}, a^{2\mathtt{L}})$ and $\tilde{\mathbf{a}} = (\tilde{a}^{1\mathtt{H}}, \tilde{a}^{2\mathtt{H}}, \tilde{a}^{1\mathtt{L}}, \tilde{a}^{2\mathtt{L}})$ be the current and the next state, respectively, with $\mathbf{a}, \tilde{\mathbf{a}} \in \{C, D\}^4$. We distinguish four cases.

1. With probability $(1-\varepsilon_{\text{IR}})^2$ no player commits an error. In that case, the transition probability from $\mathbf{a}$

to $\tilde{\mathbf{a}}$ is the same as in the baseline case, $y^{1\text{H}}_{a^{2\text{H}}a^{2\text{L}}} \cdot y^{2\text{H}}_{a^{1\text{H}}a^{1\text{L}}} \cdot y^{1\text{L}}_{a^{2\text{H}}a^{2\text{L}}} \cdot y^{2\text{L}}_{a^{1\text{H}}a^{1\text{L}}}$, with each factor $y^{ik}_{a^{\text{H}}a^{\text{L}}}$ being defined as in Eq. [8].

2. With probability $\varepsilon_{\text{IR}}(1-\varepsilon_{\text{IR}})$ player 1 commits an error but player 2 does not. In that case, the relevant transition probability is $y^{1\text{H}}_{a^{2\text{L}}a^{2\text{H}}} \cdot y^{2\text{H}}_{a^{1\text{H}}a^{1\text{L}}} \cdot y^{1\text{L}}_{a^{2\text{L}}a^{2\text{H}}} \cdot y^{2\text{L}}_{a^{1\text{H}}a^{1\text{L}}}$ (i.e., the first and the third factor are modified).

3. With the same probability $\varepsilon_{\text{IR}}(1-\varepsilon_{\text{IR}})$ player 2 commits an error but player 1 does not. The respective transition probability is $y^{1\text{H}}_{a^{2\text{H}}a^{2\text{L}}} \cdot y^{2\text{H}}_{a^{1\text{L}}a^{1\text{H}}} \cdot y^{1\text{L}}_{a^{2\text{H}}a^{2\text{L}}} \cdot y^{2\text{L}}_{a^{1\text{L}}a^{1\text{H}}}$ (the second and fourth factor are modified).

4. With probability $\varepsilon^2_{\text{IR}}$ both players commit an error simultaneously. In that case, the transition probability becomes $y^{1\text{H}}_{a^{2\text{L}}a^{2\text{H}}} \cdot y^{2\text{H}}_{a^{1\text{L}}a^{1\text{H}}} \cdot y^{1\text{L}}_{a^{2\text{L}}a^{2\text{H}}} \cdot y^{2\text{L}}_{a^{1\text{L}}a^{1\text{H}}}$ (all factors are modified compared to the baseline).

The overall transition probability from $\mathbf{a}$ to $\tilde{\mathbf{a}}$ is the weighted sum of these four conditional transition probabilities.

Equivalently, we can also write the respective transition matrix more explicitly. For a given repeated-game strategy $\mathbf{p^k} = (p^k_{CC}, p^k_{CD}, p^k_{DC}, p^k_{DD})$, define a perturbed strategy by $\tilde{\mathbf{p}}^\mathbf{k} := (p^k_{CC}, p^k_{DC}, p^k_{CD}, p^k_{DD})$ (i.e. the second and third entry change their position). For a concurrent-game strategy $\mathbf{p} = (\mathbf{p}^{\text{H}}, \mathbf{p}^{\text{L}})$, define $\tilde{\mathbf{p}} := (\tilde{\mathbf{p}}^{\text{H}}, \tilde{\mathbf{p}}^{\text{L}})$ as the strategy with perturbed components. Based on this notation, let $\mathbf{p^1}$ and $\mathbf{p^2}$ be the strategies of the two players in the same-partner treatment. Then the respective transition matrix for the case of imperfect recall can be written as

$$M_{\text{IR}} = (1-\varepsilon_{\text{IR}})^2 \cdot M(\mathbf{p^1}, \mathbf{p^2}) + \varepsilon_{\text{IR}}(1-\varepsilon_{\text{IR}})\Big(M(\tilde{\mathbf{p}}^\mathbf{1}, \mathbf{p^2}) + M(\mathbf{p^1}, \tilde{\mathbf{p}}^\mathbf{2})\Big) + \varepsilon^2_{\text{IR}} \cdot M(\tilde{\mathbf{p}}^\mathbf{1}, \tilde{\mathbf{p}}^\mathbf{2}). \qquad [16]$$

The matrices $M(\mathbf{x}, \mathbf{y})$ on the right hand side are defined as in the baseline model, Eq. [7]. In particular, as errors vanish, $\varepsilon_{\text{IR}} \to 0$, the matrix M_{IR} recovers the baseline transition matrix. For positive error rates, we can use the matrix M_{IR} in Eq. [16] to compute the invariant distribution, the players' average cooperation rates, and their expected payoffs as before.

Different-partners treatment. The logic of integrating imperfect recall into the different-partners treatment is analogous to the same-partner treatment. However, because there are now four players, there are more cases to consider. In a given round, the number of players who are subject to imperfect recall may be any number between zero and four.

Using the same notation as before, the respective transition matrix becomes

$$\begin{aligned}
M_{\text{IR}} = \;& (1-\varepsilon_{\text{IR}})^4 \quad M(\mathbf{p^1}, \mathbf{p^2}, \mathbf{p^3}, \mathbf{p^4}) \\
&+ \varepsilon_{\text{IR}}(1-\varepsilon_{\text{IR}})^3 \Big(M(\tilde{\mathbf{p}}^1, \mathbf{p^2}, \mathbf{p^3}, \mathbf{p^4}) + M(\mathbf{p^1}, \tilde{\mathbf{p}}^2, \mathbf{p^3}, \mathbf{p^4}) + M(\mathbf{p^1}, \mathbf{p^2}, \tilde{\mathbf{p}}^3, \mathbf{p^4}) + M(\mathbf{p^1}, \mathbf{p^2}, \mathbf{p^3}, \tilde{\mathbf{p}}^4)\Big) \\
&+ \varepsilon_{\text{IR}}^2(1-\varepsilon_{\text{IR}})^2 \Big(M(\tilde{\mathbf{p}}^1, \tilde{\mathbf{p}}^2, \mathbf{p^3}, \mathbf{p^4}) + M(\tilde{\mathbf{p}}^1, \mathbf{p^2}, \tilde{\mathbf{p}}^3, \mathbf{p^4}) + M(\tilde{\mathbf{p}}^1, \mathbf{p^2}, \mathbf{p^3}, \tilde{\mathbf{p}}^4) \\
&\qquad + M(\mathbf{p^1}, \tilde{\mathbf{p}}^2, \tilde{\mathbf{p}}^3, \mathbf{p^4}) + M(\mathbf{p^1}, \tilde{\mathbf{p}}^2, \mathbf{p^3}, \tilde{\mathbf{p}}^4) + M(\mathbf{p^1}, \mathbf{p^2}, \tilde{\mathbf{p}}^3, \tilde{\mathbf{p}}^4)\Big) \\
&+ \varepsilon_{\text{IR}}^3(1-\varepsilon_{\text{IR}}) \Big(M(\tilde{\mathbf{p}}^1, \tilde{\mathbf{p}}^2, \tilde{\mathbf{p}}^3, \mathbf{p^4}) + M(\tilde{\mathbf{p}}^1, \tilde{\mathbf{p}}^2, \mathbf{p^3}, \tilde{\mathbf{p}}^4) + M(\tilde{\mathbf{p}}^1, \mathbf{p^2}, \tilde{\mathbf{p}}^3, \tilde{\mathbf{p}}^4) + M(\mathbf{p^1}, \tilde{\mathbf{p}}^2, \tilde{\mathbf{p}}^3, \tilde{\mathbf{p}}^4)\Big) \\
&+ \quad \varepsilon_{\text{IR}}^4 \quad M(\tilde{\mathbf{p}}^1, \tilde{\mathbf{p}}^2, \tilde{\mathbf{p}}^3, \tilde{\mathbf{p}}^4)
\end{aligned} \qquad [17]$$

Again, given this transition matrix, it is straightforward to compute the players' cooperation rates and their payoffs, as described by Eqs. [13] and [14].

2.2.2 Behavioral spillovers

Motivation. Behavioral spillovers occur when an individual's action in one domain leads that individual to take a similar action in a different domain. Such spillovers may occur consciously or subconsciously, and they can have important policy implications (74, 75). In the context of our framework, spillovers induce additional correlations in a player's behavior across the two games. For any given history, spillovers increase the chance that a player cooperates in both games (rather than cooperating in one game and defecting in the other). Similarly, they also increase the chance that players defect in both games. In the following we describe how (exogenous) behavioral spillovers can be integrated into our framework.

Same-partner treatment. We assume spillovers occur with a constant probability ε_{SP} and they affect each player independently. Moreover, we assume that both cooperative and defective actions are equally likely to spill over to a different context. To describe the effects of such spillovers formally, we consider two players with strategies $\mathbf{p^1}$ and $\mathbf{p^2}$. We aim to describe the probability that they make the transition from state $\mathbf{a} = (a^{1\text{H}}, a^{2\text{H}}, a^{1\text{L}}, a^{2\text{L}}) \in \{C, D\}^4$ in one round to state $\tilde{\mathbf{a}} = (\tilde{a}^{1\text{H}}, \tilde{a}^{2\text{H}}, \tilde{a}^{1\text{L}}, \tilde{a}^{2\text{L}}) \in \{C, D\}^4$ in the next round. Again we need to distinguish several cases. For example, there are different ways that would lead player 1 to cooperate in both games in the next round:

1. Player 1 may decide to cooperate in both games from the outset. Given the previous actions of the second player, this happens with probability $p^{1\text{H}}_{a^{2\text{H}}a^{2\text{L}}} \cdot p^{1\text{L}}_{a^{2\text{H}}a^{2\text{L}}}$.

2. Player 1 initially decides to cooperate in the high game but to defect in the other; but due to a spillover, the player ends up cooperating in both games. The respective probability is $\frac{\varepsilon_{\text{SP}}}{2} \cdot p^{1\text{H}}_{a^{2\text{H}}a^{2\text{L}}}(1-p^{1\text{L}}_{a^{2\text{H}}a^{2\text{L}}})$. The factor of one half indicates that spillovers could equally lead the player to defect in both games.

3. Similarly, player 1 may wish to defect in the high game, to cooperate in the low game, but ends up cooperating on both games due to a spillover. This probability is $\frac{\varepsilon_{\text{SP}}}{2} \cdot (1-p^{1\text{H}}_{a^{2\text{H}}a^{2\text{L}}})p^{1\text{L}}_{a^{2\text{H}}a^{2\text{L}}}$.

4. If player 1 wishes to defect in both games, spillovers cannot affect the player's decision.

Overall, in the presence of spillovers, the transition probability from state $\mathbf{a}$ to $\tilde{\mathbf{a}}$ takes the form

$$m_{\mathbf{a},\tilde{\mathbf{a}}} = z^1_{a^{2\mathrm{H}}a^{2\mathrm{L}}} \cdot z^2_{a^{1\mathrm{H}}a^{1\mathrm{L}}}. \qquad [18]$$

Here the two variable $z^i_{a^{\mathrm{H}}a^{\mathrm{L}}}$ for $i \in \{1,2\}$ describe the decisions of the two players in both the high and the low game. For example, for the first variable,

$$z^1_{a^{2\mathrm{H}},a^{2\mathrm{L}}} := \begin{cases} p^{1\mathrm{H}}_{a^{2\mathrm{H}}a^{2\mathrm{L}}} p^{1\mathrm{L}}_{a^{2\mathrm{H}}a^{2\mathrm{L}}} + \frac{\varepsilon_{\mathrm{SP}}}{2}\Big[p^{1\mathrm{H}}_{a^{2\mathrm{H}}a^{2\mathrm{L}}}(1-p^{1\mathrm{L}}_{a^{2\mathrm{H}}a^{2\mathrm{L}}}) + (1-p^{1\mathrm{H}}_{a^{2\mathrm{H}}a^{2\mathrm{L}}})p^{1\mathrm{L}}_{a^{2\mathrm{H}}a^{2\mathrm{L}}}\Big] & \text{if } \tilde{a}^{1\mathrm{H}}=C,\ \tilde{a}^{2\mathrm{H}}=C \\ (1-\varepsilon_{\mathrm{SP}})p^{1\mathrm{H}}_{a^{2\mathrm{H}}a^{2\mathrm{L}}}(1-p^{1\mathrm{L}}_{a^{2\mathrm{H}}a^{2\mathrm{L}}}) & \text{if } \tilde{a}^{1\mathrm{H}}=C,\ \tilde{a}^{2\mathrm{H}}=D \\ (1-\varepsilon_{\mathrm{SP}})(1-p^{1\mathrm{H}}_{a^{2\mathrm{H}}a^{2\mathrm{L}}})p^{1\mathrm{L}}_{a^{2\mathrm{H}}a^{2\mathrm{L}}} & \text{if } \tilde{a}^{1\mathrm{H}}=D,\ \tilde{a}^{2\mathrm{H}}=C \\ (1-p^{1\mathrm{H}}_{a^{2\mathrm{H}}a^{2\mathrm{L}}})(1-p^{1\mathrm{L}}_{a^{2\mathrm{H}}a^{2\mathrm{L}}}) + \frac{\varepsilon_{\mathrm{SP}}}{2}\Big[p^{1\mathrm{H}}_{a^{2\mathrm{H}}a^{2\mathrm{L}}}(1-p^{1\mathrm{L}}_{a^{2\mathrm{H}}a^{2\mathrm{L}}})+(1-p^{1\mathrm{H}}_{a^{2\mathrm{H}}a^{2\mathrm{L}}})p^{1\mathrm{L}}_{a^{2\mathrm{H}}a^{2\mathrm{L}}}\Big] & \text{if } \tilde{a}^{1\mathrm{H}}=D,\ \tilde{a}^{2\mathrm{H}}=D \end{cases} \qquad [19]$$

The variable $z^2_{a^{1\mathrm{H}},a^{1\mathrm{L}}}$ for the second player is defined analogously. In the limiting case that exogenous spillovers are rare, $\varepsilon_{\mathrm{SP}} = 0$, the transition probability [18] simplifies to the formula [7] of the baseline model, which is reassuring. However, as $\varepsilon_{\mathrm{SP}}$ increases, each player becomes increasingly unlikely to choose different actions in the two games. In particular, in the limiting case $\varepsilon_{\mathrm{SP}} = 1$, there is a perfect correlation between a player's actions. Each player either cooperates in both games, or defects in both games.

By collecting the transition probabilities [18] and writing them as a matrix $M_{\mathrm{SP}} = (m_{\mathbf{a},\tilde{\mathbf{a}}})$, we can again use the methods from the baseline model to compute expected cooperation rates and payoffs.

Different-partners treatment. Spillovers can be incorporated in the same way as in the same-partners treatment. If $\mathbf{p^1}$, $\mathbf{p^2}$, $\mathbf{p^3}$, $\mathbf{p^4}$ are the strategies of the four players, and $\mathbf{a}$ and $\tilde{\mathbf{a}}$ are the current and the next state, respectively, the transition probability takes the form

$$m_{\mathbf{a},\tilde{\mathbf{a}}} = z^1_{a^{2\mathrm{H}}a^{3\mathrm{L}}} \cdot z^2_{a^{1\mathrm{H}}a^{4\mathrm{L}}} \cdot z^3_{a^{4\mathrm{H}}a^{1\mathrm{L}}} \cdot z^4_{a^{3\mathrm{H}}a^{2\mathrm{L}}}. \qquad [20]$$

The individual factors $z^i_{a^{\mathrm{H}}a^{\mathrm{L}}}$ are defined analogously as in the previous section, see Eq. [19]. Based on the resulting transition matrix $M_{\mathrm{SP}} = (m_{\mathbf{a},\tilde{\mathbf{a}}})$, we can again compute cooperation rates and payoffs.

2.2.3 Preferential updating

Motivation. The two previous subsections dealt with two plausible kinds of errors. These errors affect how people with given strategies act in a concurrent game. The next two subsections describe two behaviorally plausible modifications of the learning process. These modifications affect how people choose their strategies in the first place. The baseline model assumes that in each step of the learning process, players are equally likely to update their strategy for the high game and for the low game. In the following, we discuss a model extension that allows for preferential updating. Here, players are more likely to update the strategy

in the game that currently yields the lower relative payoff.

Implementation of the learning process. We incorporate preferential updating as follows. As in the baseline model, we assume that in any given time step t, one individual, say player j, is picked at random. This player is then given a chance to generate an alternative strategy, by either modifying their strategy in the high or the low game. However, in this case, the high-game strategy is modified with a probability given by the Fermi-function,

$$\xi^j = \frac{1}{1+\exp[\kappa(\frac{\pi^{j\texttt{H}}}{b_\texttt{H}} - \frac{\pi^{j\texttt{L}}}{b_\texttt{L}})]}. \qquad [21]$$

The parameter $\kappa \geq 0$ measures to which extent updating is biased towards one game or the other. For $\kappa = 0$, we obtain $\xi^j = 1/2$, irrespective of the value of the other variables. In that case, we recover the baseline model where both games are equally likely to be modified. For positive κ, the player is more likely to modify the strategy that currently yields a lower payoff, relative to the maximum achievable payoff b_k in the respective game. Once an alternative strategy is generated, the further process is the same as described in the main text. As described there, the player is more likely to adopt the generated strategy $\tilde{\mathbf{p}}^{\mathbf{j}}$, the higher the hypothetical payoff $\tilde{\pi}^j$ is compared to the player's current payoff π^j. If $\tilde{\mathbf{p}}^{\mathbf{j}}$ yields a high payoff, it is likely to be adopted. If $\tilde{\mathbf{p}}^{\mathbf{j}}$ yields a low payoff, it is likely to be discarded and the player continues to use their current strategy $\mathbf{p}^{\mathbf{j}}$.

2.2.4 Updating under narrow bracketing

Motivation. Narrow bracketing refers to situations in which people make decisions in one domain without fully taking into account its consequences on decisions in other domains (76). Such a bias may also unfold in concurrent games. Players who update their strategies for a given repeated game $k \in \{\texttt{H}, \texttt{L}\}$ may neglect the impact of this strategy change on the other game. As a result, a strategy change that is beneficial in the respective game may be detrimental overall. In the following, we describe a simple model of narrow bracketing in the context of concurrent games.

Implementation of the learning process. The learning process under narrow bracketing follows along the same lines as the baseline learning process described in the main text. As before, a randomly chosen focal player updates its strategy in a randomly chosen game $k \in \{\texttt{H}, \texttt{L}\}$. When deciding whether to adopt the new concurrent strategy $\tilde{\mathbf{p}}^{\mathbf{j}}$, with probability $1 - \lambda$, the player compares the total payoffs $\tilde{\pi}^j$ and π^j. With the converse probability λ, however, the player only compares the payoffs $\tilde{\pi}^{jk}$ and π^{jk} of the particular game k in which the strategy $\tilde{\mathbf{p}}^j$ got updated. In particular, a player may adopt the alternative strategy because $\tilde{\pi}^{jk} > \pi^{jk}$ even though the total payoff effect is negative, $\tilde{\pi}^j = \tilde{\pi}^{j\texttt{H}} + \tilde{\pi}^{j\texttt{L}} < \pi^{j\texttt{H}} + \pi^{j\texttt{L}} = \pi^j$. The total effect may be negative because in concurrent games, a player's strategy in one game can affect the co-player's response in the other game. We refer to λ as the probability of narrow bracketing. When $\lambda = 0$, decisions are made as in the baseline model. As λ increases, updating decisions are increasingly based on the payoff

effects in repeated game k only.

2.3 Experimental methods

2.3.1 Experimental procedures

Experimental setup. Our experiments has three different treatments, matching those of the theoretical model **Fig. 1**. The treatments differ in the number of games played simultaneously and in the number of co-players. The control treatment is a standard 2-player game. The same-partner treatment is also a 2-player game, but now both players interact through two games simultaneously. The different-partners treatment also involves two games, but now each participant interacts with two different co-players, one for each game.

**Treatment**	**Number of games**	**Number of co-players**
Control	1	1
Same-Partner	2	1
Different-Partner	2	2

Each stage game is a donation game. Players choose between paying a cost to send a benefit to the co-player or do nothing. The games differ only in the benefit b sent to the other player; the cost c remains constant. In the high game, $b = 4$ points and $c = 2$ points, whereas in the low game, $b = 3$ points and $c = 2$ points.

Participants interacted for a minimum of 20 rounds with their respective co-player(s). To avoid end-game effects, after the 20th round, each subsequent round had a 50% chance of occurring. Participants in the multi-game treatments (same-partner and different-partners) made decisions for both games simultaneously (i.e. on the same page) on every round. The decisions for each game were separately elicited from each other. Participants were told what the other player(s) had chosen after every round, and they were reminded of their co-players' previous decisions when making their next one. Once all rounds had been played, each participant was informed of their total payoff across the whole game in points as well as the converted amount (in GBP). They were asked to fill a demographics form, were thanked for their time and informed that the money would be paid to them by the lab manager at a future date. The games are built with the python package oTree (77) and ran online. oTree was developed specifically to run real-time interactive sessions with multiple participants.

Participant recruitment. We recruited 363 participants between April and October 2021 from the University of Exeter pool of subjects. Participants received a baseline payment of £3 for participating in our experiment. In addition, they were able to receive a bonus payment based on their performance during the experiment. To earn this bonus, participant accumulated points from one or two games that were converted to British pounds (£) at the end. One point was worth £1/75 (or 20 points = £0.26) and the average

bonus earned was £1.66, £1.62, and £0.88 in the Different-Partners, Same-partner and Control treatments, respectively. The sample size was estimated from a rough power analysis and based on previous similar research, given constraints on the size of the FEELE subject pool. The student pool showed lower dropout rates than most online recruiting platforms. The experiment was approved by the faculty of Medicine of the Christian-Albrechts-Univeristy in Kiel (ID number D 571/20)

Experimental procedures. The design of the experiment was between-subjects, meaning that participants only took part in one treatment. Fifteen sessions of up to 32 participants were run sequentially. Each session was for one treatment only. Participants signed up for an open slot on the FEELE platform of the University of Exeter without prior knowledge of the experiment or treatment. Ten minutes prior to the start of the session, the experimenter opened a room in oTree so that participants could connect and wait until everyone had arrived. Once the room was full, the experimenter started the study. Once participants entered the study, the first page was a consent form describing their rights. Clicking on the button to continue was explained to mean they gave their consent to take part in the study. Then the instructions were provided along with a series of comprehension questions. If they gave a wrong answer, a message appeared on the screen asking them to reread the instructions and change their answer. They could only continue once they gave the correct answer to each question. After these steps were completed, participants entered a virtual waiting room.

Once at least four participants were in the virtual waiting room, they would be paired together to form a group and proceed to the main task. Players were always grouped together in fours, but they only play as an interactive group of four in the different-partners treatment. This constraint comes from the design of the different-partners treatment, but to ensure consistency in waiting times throughout the experiment, it was implemented for all treatments. In the other two treatments, two pairs go through the game at the same time but are unaware of the other pair's existence. Participants waited for a maximum of seven minutes. If not enough players showed up in that timeframe, participants were considered unmatched and instructed to leave the study and receive their participation fee, but no bonus. If after pairing one player in a group dropped out, all others were informed and asked to leave the study. They were paid a fixed bonus of £1.

2.3.2 Statistical methods

For our data analysis, we only considered groups where all participants completed their total number of rounds, resulting in 316 subjects in 79 groups. To increase power, in the same-partner treatment, we separate the two independent pairs into subgroups. We use these smaller denominations for our group level analysis. This means we have 36 groups of 2 for the same-partner treatment, 32 groups of 4 for the different partners treatment, and 29 groups of 4 for the control. Since all considered groups played at least 20 rounds but differed in the total number of rounds, we used only rounds 1-20 for the main analysis. All averages are aggregated at the group level for all our analysis so that we get the average cooperation across all rounds of interest and all group participants.

2.3.3 Additional results

Overall cooperation levels in the two multi-game treatment do not differ significantly despite difference in conditional behavior, suggesting that the difference in overall cooperation rates is not due to reciprocity. In addition, cooperation in both multi-game treatments is lower the first round (From 79% in control to 64% in the same-partner treatment and 66% in the different-partners treatment in the high game, and from 67% in control to 49% in the same-partner treatment and 55% in the different-partners treatment in the low game). Here we report the statistical results to test if this difference is significant already on the first round of the game(s). The results are mixed, and we only find a significant difference in the high game for the same-partner treatment $p = 0.021, \delta = 0.33$ $(95\%\ CI[0.02, 0.57])$, and in the low game for the different-partner treatment $p = 0.024, \delta = 0.29$ $(95\%\ CI[0.04, 0.51])$. (see **Fig. 4**.)

3 Supplementary Figures

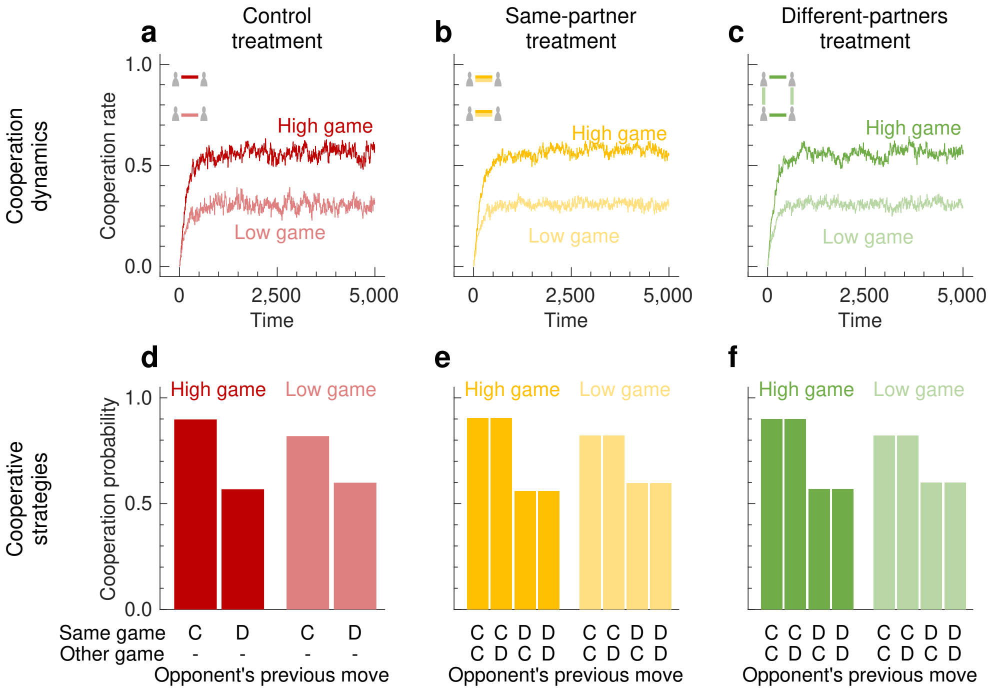

Figure S1: Learning dynamics among players who treat each game as independent. This figure uses the same setup and the same parameters as in **Fig. 2**. Panels **a-c** show the overall cooperation rates for each treatment, and panels **d-f** show the conditional strategies for each treatment (probability to cooperate based on the previous actions). However, this time, players in the same-partner and the different-partners treatment are artificially restricted to treat each repeated game as independent. That is, for these simulations, we only allow those mutant strategies that satisfy Eq. [3] in the main text. In this case, all three treatments are equivalent. They lead to the same cooperation dynamics, and they generate the same cooperative strategies.

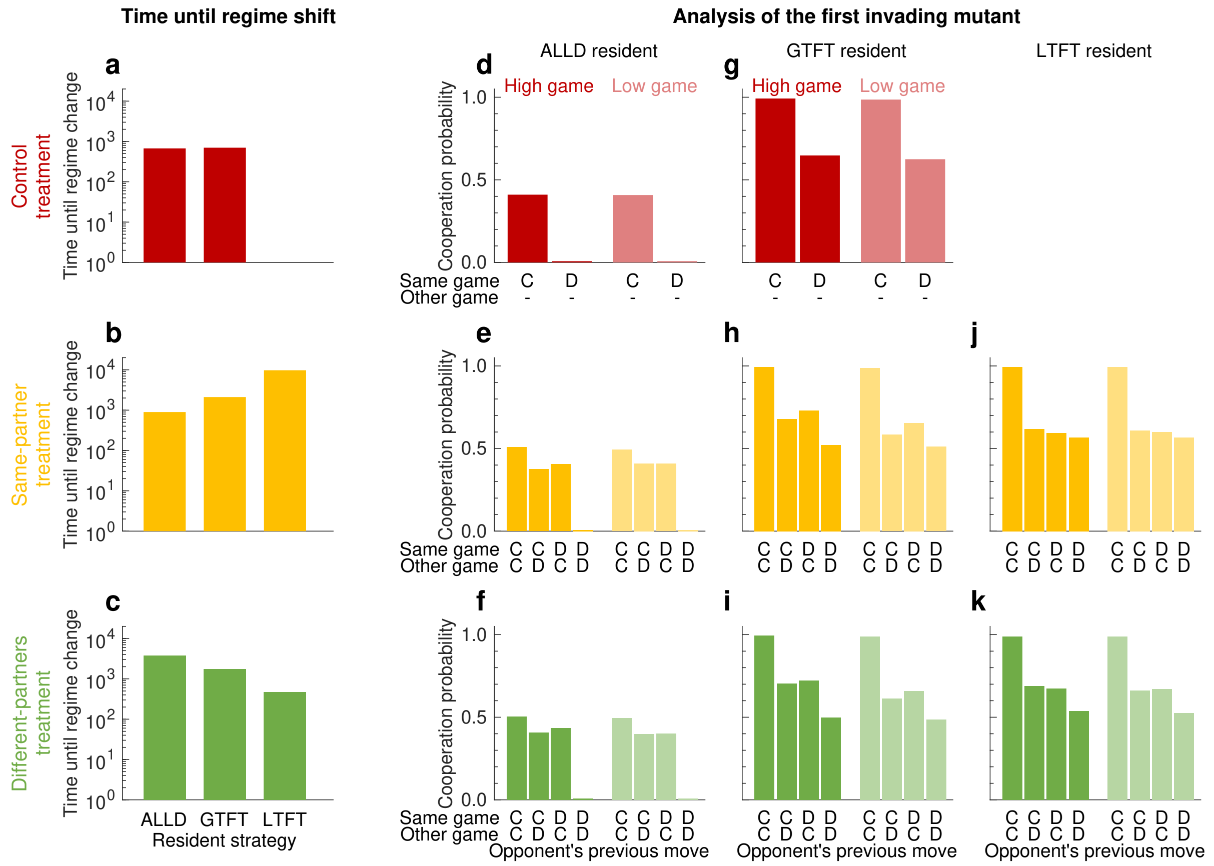

Figure S2: Invasion dynamics across the three treatments. To gain further insights into the learning dynamics, we have run simulations assuming that players start out with predefined strategies. Here, we allow for three such strategies. `ALLD` cooperates with probability 0.01 each round, corresponding to a noisy variant of always defect. `GTFT` treats each game as independent and cooperates with probability 0.99 after cooperation, and 0.50 after defection. `LTFT` corresponds to a version of `GTFT` with linkage. It cooperates with probability 0.99 if the co-player previously cooperated in both games; otherwise it cooperates with with probability 0.50. Note that this strategy only exists in the same-partner and the different-partners treatment. Initially, all players are assumed to adopt the same strategy (we refer to the strategy as the 'resident'). Then we simulate the learning dynamics until there has been a 'regime shift' (meaning that the average cooperation rate among the four players exceeds 50% after starting from `ALLD`, or that it falls below 50% after starting from `GTFT` or `LTFT`). We record the time it takes for a regime shift to occur (panels **a –c**). In addition, we record the first strategy that a player adopts instead of the resident strategy (to which we refer as the 'first invading mutant', panels **d–k**). In the same-partner treatment, `LTFT` is the most robust resident strategy with respect to regime shifts. In the different-partners treatment, the most robust strategy is `ALLD`. In general, we find that `ALLD` is typically invaded by conditionally cooperative strategies (which show a very small cooperation probability after full defection). In contrast, both `GTFT` and `LTFT` are typically invaded by strategies similar to `LTFT` in the last two treatments. All bars represent averages of 1,000 independent simulations, using the parameters of **Fig. 2**.

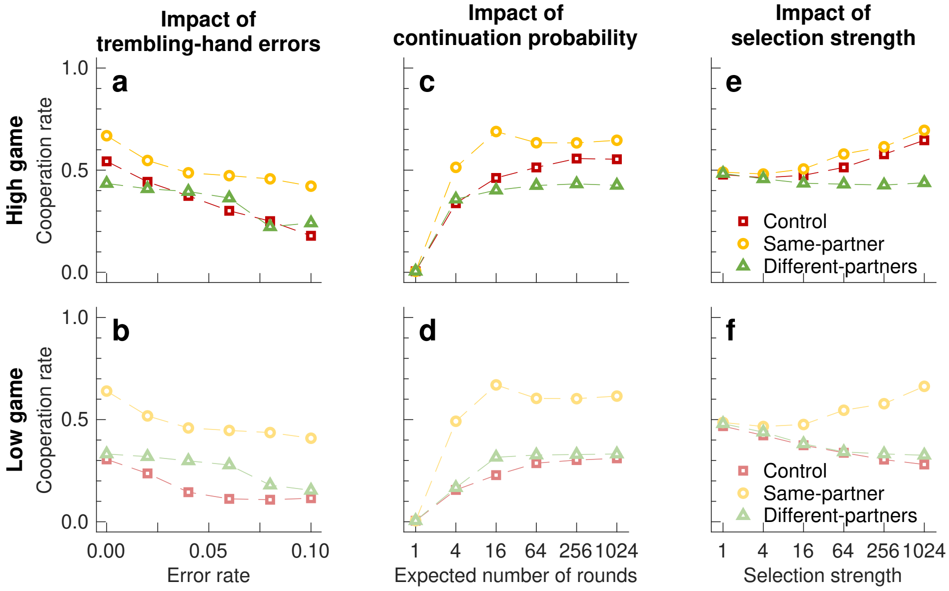

Figure S3: Robustness of evolutionary results. To explore the robustness of our results, we systematically vary three key parameters. **a,** In previous figures, we have assumed that players always perfectly implement their intended actions. Here, we explore the impact of trembling-hand errors, see Section 2.1.4. Overall, such errors make it more difficult to sustain cooperation. **b,** In our previous theoretical analysis, we have assumed for simplicity that the game is infinitely repeated. However, it is straightforward to analyze repeated games in which after each round, the game only continues with a probability $\delta < 1$, see for example Ref. (78). Here, we report the corresponding simulation results, as a function of the expected number of rounds, $1/(1-\delta)$. As one may expect, players become more cooperative when there are more rounds. **c,** Finally, we have also varied the selection strength of the evolutionary process (in the main text we use $\beta = 200$). The stronger selection, the more we see a difference between the three treatments. Across all conditions, our results suggest that one should expect most cooperation in the same-partner treatment, in line with Ref. (47). Parameters are the same as in **Fig. 2**.

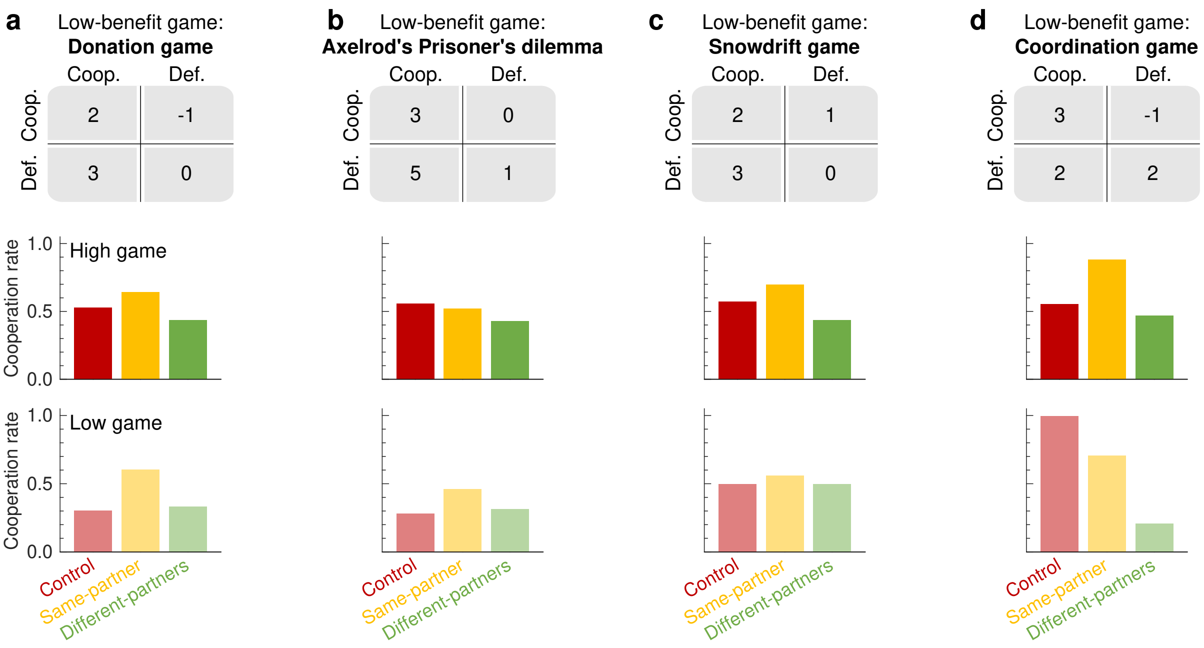

Figure S4: Concurrent games beyond the prisoner's dilemma. a, In our previous analysis, we assumed players engage in different donation games. The respective result of **Fig. 2** is reproduced here. In addition, we consider three cases in which the low-benefit game is replaced by **b,** the payoffs used by Axelrod (23), **c,** a snowdrift game, and **d,** a coordination game. Across all games, we observe that the same partner-treatment should result in more cooperation than the different-partners treatment. Yet in the case of a coordination game, the control treatment seems to be best able to single out the most efficient equilibrium. Except for the low-game payoffs, all parameters are as in **Fig. 2**.

	Dependent variable:	
	Cooperation	
	High game	Low game
Partner's previous decision in the high game	0.673^{***}	
	(0.043)	
Partner's previous decision in the low game		0.651^{***}
		(0.034)
Constant	0.222^{***}	0.216^{***}
	(0.034)	(0.025)
Observations	2,470	2,470
R^2	0.452	0.424
Adjusted R^2	0.452	0.424
Residual Std. Error (df = 2468)	0.344	0.369
Note:	*p<0.05; **p<0.01; ***p<0.001	

Table S1: Conditional cooperation in the control treatment. Similar to **Table 1**, here we report the results of a linear regression for the players' conditional cooperation probability in the control treatment. Based on the data of our behavioral experiment, we estimate how likely participants are to cooperate, depending on their partner's previous behavior. We observe that previous decisions have a significant impact on the probability of cooperation in the current round (in the high game: $p < 0.001$, in the low game: $p < 0.001$).

Instructions

You are **Participant 1** in this study. The other participants are **Participant 2** and **Participant 3**.

In each round, you and Participant 2 will simultaneously interact with each other in Task A, and you and Participant 3 will simultaneously interact with each other in Task B. In each task, you and your co-participant on that task must choose between two options. The points you earn in each round will depend on what you, Participant 2 and Participant 3 independently decide for your respective tasks. The two tasks are independent: The points you earn in either task do not depend on the decisions made in the other. In the beginning of each round, you and the other two participants receive an endowment of 2 points in task A and 2 points in task B.

Below you can see the two options that **you and Participant 2** can choose from in Task A in each round.

Task A:

- You **lose 2** pts for Participant 2 to **receive 4** pts
- You **lose 0** pts for Participant 2 to **receive 0** pts

Participant 2 faces exactly the same Task A with **you** as the recipient. You and Participant 2 have to choose simultaneously between these two options.

Below you can see the two options that that **you and Participant 3** can choose from in Task B in each round.

Task B:

- You **lose 2** pts for Participant 3 to **receive 3** pts
- You **lose 0** pts for Participant 3 to **receive 0** pts

Participant 3 faces exactly the same Task B with **you** as the recipient. You and Participant 3 have to choose simultaneously between these two options.

Note that, while Participant 2 and Participant 3 interact with you in Tasks A and B, they both also simultaneously interact with another Participant (but not with each other) in a similar task. That is, each of you is engaged in two tasks simultaneously at all times.

The session will last for multiple rounds. There will be at least 20 rounds. After the 20th round, there will be a 50% chance of another round. After that round, there will again be a 50% chance of another round, and after that round, there will again be another 50% chance of another round, and so on until the tasks end.

You and the other participants are both endowed with 80 points at the start of the game (2 points for Task A and 2 points for Task B, for each of the 20 rounds). For any additional round that may occur after the 20th round, you receive an additional endowment of 2 points for Task A and 2 points for Task B.

Please answer the following questions to continue:

In Task A, what amount will you receive from Participant 2 if they choose to pay 2 pts:

- ○ You will earn 0 pts.
- ○ You will earn 2 pts.
- ● You will earn 4 pts.

What are the chances that there will be another round after the 20th round?

- ○ 10%
- ● 50%
- ○ 100%

What are the chances that there will be another round after the 21th round?

- ○ 10%
- ● 50%
- ○ 100%

Next

Example

Recall the options for Task A and Task B:

Task A:
(played with Participant 2)

- You **lose 2 pts** for Participant 2 to **receive 4 pts**.
- You **lose 0 pts** for Participant 2 to **receive 0 pts**.

Task B:
(played with Participant 3)

- You **lose 2 pts** for Participant 3 to **receive 3 pts**.
- You **lose 0 pts** for Participant 3 to **receive 0 pts**.

Example

Consider a scenario where Participant 1 and Participant 2 made the following decisions:

Task A:
(played with Participant 2)

Participant 1 chose:

- to **lose 2 pts** for Participant 2 to **receive 4 pts**

Participant 2 chose:

- to **lose 0 pts** for Participant 1 to **receive 0 pts**

Participant 1's points in this round: -2 + 0 = **-2**

Participant 2's points in this round: 0 + 4 = **4**

Consider a scenario where Participant 1 and Participant 3 made the following decisions:

Task B:
(played with Participant 3)

Participant 1 chose:

- to **lose 2 pts** for Participant 3 to **receive 3 pts**

Participant 3 chose:

- to **lose 2 pts** for Participant 1 to **receive 3 pts**

Participant 1's points in this round: -2 + 3 = **1**

Participant 3's points in this round: -2 + 3 = **1**

In total across both tasks, Participant 1 earned **-1 pts**.

Please answer the following questions to continue:

Across both tasks, how many points did Participant 1 earn in total?

- (●) -1 points
- ( ) 1 points
- ( ) 5 points

In Task A, how many points did Participant 2 earn?

- ( ) 0 points
- ( ) 2 points
- (●) 4 points

In Task B, how many points did Participant 3 earn?

- ( ) -2 points
- (●) 1 points
- ( ) 3 points

Next

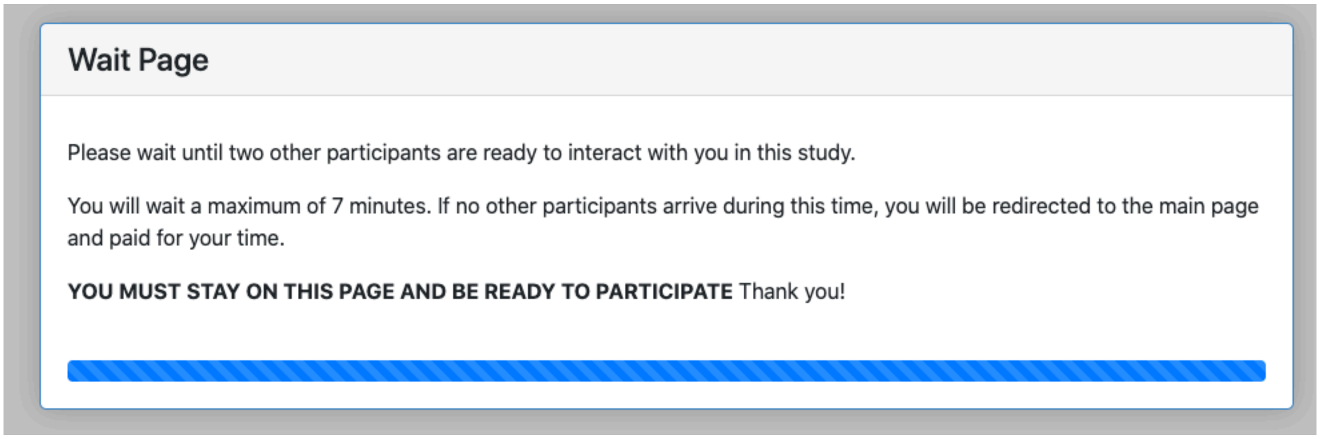
Wait Page

Please wait until two other participants are ready to interact with you in this study.

You will wait a maximum of 7 minutes. If no other participants arrive during this time, you will be redirected to the main page and paid for your time.

YOU MUST STAY ON THIS PAGE AND BE READY TO PARTICIPATE Thank you!

Round Decision

In the previous round:

Task A:
(played with Participant 2)

You chose:

to **pay 2 pts** for Participant 2 to **receive 4 pts**.

Participant 2 chose:

to **pay 2 pts** for you to **receive 4 pts**.

Task B:
(played with Participant 3)

You chose:

to **pay 2 pts** for Participant 3 to **receive 3 pts**.

Participant 3 chose:

to **pay 0 pts** for you to **receive 0 pts**

In this round:

Below are your two options for **Task A** and **Task B**, respectively. You need to make a choice in each task to continue.

Task A:

(played with Participant 2)

- (●) You lose 2 pts for Participant 2 to receive 4 pts.
- ( ) You pay 0 pts for Participant 2 to receive 0 pts.

Task B:

(played with Participant 3)

- ( ) You lose 2 pts for Participant 3 to receive 3 pts.
- (●) You pay 0 pts for Participant 3 to receive 0 pts.

Next

Wait Page

Please wait while the other participants make their decision.

If the waiting time is longer than 5 minutes the study will assume the other player dropped out. If this happens you'll be notified and paid a small bonus for your time.

Please don't leave before you are notified to.

Round Results

Task A:

(played with Participant 2)

You chose:

- to **lose 2 pts** for Participant 2 to **receive 4 pts**.

Participant 2 chose:

- to **lose 2 pts** for you to **receive 4 pts**.

Your points in this round: -2 + 4 = **2**

Task B:

(played with Participant 3)

You chose:

- to **lose 0 pts** for Participant 3 to **receive 0 pts**.

Participant 3 chose:

- to **lose 0 pts** for you to **receive 0 pts**.

Your points in this round: 0 + 0 = **0**

Next

Final results

Task A:

Round	My outcome	Opponent's outcome
1	4 pts	4 pts
2	4 pts	4 pts
3	4 pts	4 pts
4	4 pts	4 pts
5	4 pts	4 pts
6	4 pts	4 pts
7	4 pts	4 pts
8	4 pts	4 pts
9	4 pts	4 pts
10	4 pts	4 pts
11	4 pts	4 pts
12	4 pts	4 pts
13	4 pts	4 pts
14	4 pts	4 pts
15	4 pts	4 pts
16	4 pts	4 pts
17	4 pts	4 pts
18	4 pts	4 pts
19	4 pts	4 pts
20	4 pts	4 pts
21	4 pts	4 pts
22	4 pts	4 pts

In Task A, across all rounds, you earned a total of **88** points.

Task B:

Round	My outcome	Opponent's outcome
1	0 pts	5 pts
2	3 pts	3 pts
3	3 pts	3 pts
4	3 pts	3 pts
5	3 pts	3 pts
6	3 pts	3 pts
7	3 pts	3 pts
8	3 pts	3 pts
9	3 pts	3 pts
10	3 pts	3 pts
11	3 pts	3 pts
12	3 pts	3 pts
13	3 pts	3 pts
14	3 pts	3 pts
15	3 pts	3 pts
16	3 pts	3 pts
17	3 pts	3 pts
18	3 pts	3 pts
19	3 pts	3 pts
20	3 pts	3 pts
21	3 pts	3 pts
22	3 pts	3 pts

In Task B, across all rounds, you earned a total of **63** points.

Next

Demographic Questions

Please answer the following questions. These are used for demographic purposes only. This information will not be associated with your name. You remain anonymous to all the other players and the experimenters.

What is your age?

What gender do you identify as?

- ◯ Female
- ◯ Male
- ◯ Other

What is the total combined income of your household?

- ◯ £9.999 or below
- ◯ £10.000 - £29.999
- ◯ £30.000 - £49.999
- ◯ £50.000 - £69.999
- ◯ £70.000 - £89.999
- ◯ £90.000 or over
- ◯ Prefer not to say

What is the highest level of education you have completed?

- ◯ No formal education
- ◯ GCSE or equivalent
- ◯ A-Levels or equivalent
- ◯ Vocational training
- ◯ Undergraduate degree
- ◯ Postgraduate degree
- ◯ Prefer not to say

What is your ethnicity?

- ◯ Asian/Asian British
- ◯ Black/African/Caribbean/Black British
- ◯ Mixed/Multiple Ethnic groups
- ◯ White
- ◯ Other

Next

Quick Survey

We appreciate feedback. If you have any comments on the implementation of this study, or on the user interface, please write it down below.

Next

The End of the Study

The study is now over. **Thank you for participating!** Below is your total score and the money you earned.

**Total points earned in all rounds across both tasks**	88 + 68 = 156
**Conversion rate**	15pts = £0.20
**Total of the bonus in GBP**	£2.08
**Participation payment**	£3.00

**Final Payment to you (participation payment + bonus)**	£5.08

You can now close your browser.

You will be paid directly into your student bank account by the University of Exeter Finance Department. Please ensure your bank account details are up to date in your SRS account. Payment usually takes 1 to 2 weeks to come through.

4 Supplementary References

[1] Hofbauer, J. & Sigmund, K. *Evolutionary Games and Population Dynamics* (Cambridge University Press, Cambridge, UK, 1998).

[2] Nowak, M. A. *Evolutionary dynamics* (Harvard University Press, Cambridge MA, 2006).

[3] Sigmund, K. The calculus of selfishness. In *The Calculus of Selfishness* (Princeton University Press, 2010).

[4] Broom, M. & Rychtář, J. *Game-Theoretical Models in Biology* (Chapman and Hall/CRC, 2013).

[5] Traulsen, A. & Hauert, C. Stochastic evolutionary game dynamics. In Schuster, H. G. (ed.) *Reviews of Nonlinear Dynamics and Complexity*, 25–61 (Wiley-VCH, Weinheim, 2009).

[6] Rand, D. G. & Nowak, M. A. Human cooperation. *Trends in cognitive sciences* **17**, 413–425 (2013).

[7] Perc, M., Gómez-Gardeñes, J., Szolnoki, A., Floría, L. M. & Moreno, Y. Evolutionary dynamics of group interactions on structured populations: A review. *Journal of The Royal Society Interface* **10**, 20120997 (2013).

[8] Perc, M. *et al.* Statistical physics of human cooperation. *Physics Reports* **687**, 1–51 (2017).

[9] Hilbe, C., Chatterjee, K. & Nowak, M. A. Partners and rivals in direct reciprocity. *Nature Human Behaviour* **2**, 469–477 (2018).

[10] García, J. & van Veelen, M. No strategy can win in the repeated prisoner's dilemma: Linking game theory and computer simulations. *Frontiers in Robotics and AI* **5**, 102 (2018).

[11] Cressman, R., Gaunersdorfer, A. & Wen, J. F. Evolutionary and dynamic stability in symmetric evolutionary games with two independent decisions. *International Game Theory Review* **2** (2000).

[12] Chamberland, M. & Cressman, R. An example of dynamic (in)consistency in symmetric extensive form evolutionary games. *Games and Economic Behavior* **30**, 319–326 (2000).

[13] Hashimoto, K. Unpredictability induced by unfocused games in evolutionary game dynamics. *Journal of Theoretical Biology* **241**, 669–675 (2006).

[14] Venkateswaran, V. R. & Gokhale, C. S. Evolutionary dynamics of complex multiple games. *Proceedings of the Royal Society B* **286**, 20190900 (2019).

[15] Sigmund, K., Hauert, C. & Nowak, M. A. Reward and punishment. *Proceedings of the National Academy of Sciences USA* **98**, 10757–10762 (2001).

[16] Boyd, R., Gintis, H., Bowles, S. & Richerson, P. The evolution of altruistic punishment. *Proceedings of the National Academy of Sciences USA* **100**, 3531–3535 (2003).

[17] Panchanathan, K. & Boyd, R. Indirect reciprocity can stabilize cooperation without the second-order free-rider problem. *Nature* **432**, 499–502 (2004).

[18] Fowler, J. H. Altruistic punishment and the origin of cooperation. *Proceedings of the National Academy of Sciences USA* **102**, 7047–7049 (2005).

[19] dos Santos, M., Rankin, D. J. & Wedekind, C. The evolution of punishment through reputation. *Proceedings of the Royal Society B* **278**, 371–377 (2011).

[20] Hauser, O. P., Hendriks, A., Rand, D. G. & Nowak, M. A. Think global, act local: Preserving the global commons. *Scientific Reports* **6**, 36079 (2016).

[21] Pal, S. & Hilbe, C. Reputation effects drie the joint evolution of cooperation and social rewarding. *Nature Communications* **13**, 5928 (2022).

[22] Trivers, R. L. The evolution of reciprocal altruism. *The Quarterly Review of Biology* **46**, 35–57 (1971).

[23] Axelrod, R. & Hamilton, W. D. The evolution of cooperation. *Science* **211**, 1390–1396 (1981).

[24] Boyd, R. & Lorberbaum, J. P. No pure strategy is evolutionarily stable in the repeated prisoner's dilemma game. *Nature* **327**, 58–59 (1987).

[25] Nowak, M. & Sigmund, K. A strategy of win-stay, lose-shift that outperforms tit-for-tat in the prisoner's dilemma game. *Nature* **364**, 56–58 (1993).

[26] Frean, M. R. The prisoner's dilemma without synchrony. *Proceedings of the Royal Society B* **257**, 75–79 (1994).

[27] Hauert, C. & Schuster, H. G. Effects of increasing the number of players and memory size in the iterated prisoner's dilemma: a numerical approach. *Proceedings of the Royal Society B* **264**, 513–519 (1997).

[28] Szabó, G., Antal, T., Szabó, P. & Droz, M. Spatial evolutionary prisoner's dilemma game with three strategies and external constraints. *Physical Review E* **62**, 1095–1103 (2000).

[29] Killingback, T. & Doebeli, M. The continuous Prisoner's Dilemma and the evolution of cooperation through reciprocal altruism with variable investment. *The American Naturalist* **160**, 421–438 (2002).

[30] Nowak, M. A., Sasaki, A., Taylor, C. & Fudenberg, D. Emergence of cooperation and evolutionary stability in finite populations. *Nature* **428**, 646–650 (2004).

[31] Kurokawa, S., Wakano, J. & Ihara, Y. Generous cooperators can outperform non-generous cooperators when replacing a population of defectors. *Theoretical Population Biology* **77** (2010).

[32] Martinez-Vaquero, L. A., Cuesta, J. A. & Sanchez, A. Generosity pays in the presence of direct reciprocity: A comprehensive study of 2x2 repeated games. *PLoS ONE* **7**, E35135 (2012).

[33] Grujic, J., Cuesta, J. A. & Sanchez, A. On the coexistence of cooperators, defectors and conditional cooperators in the multiplayer iterated prisoner's dilemma. *Journal of Theoretical Biology* **300**, 299–308 (2012).

[34] van Veelen, M., García, J., Rand, D. G. & Nowak, M. A. Direct reciprocity in structured populations. *Proceedings of the National Academy of Sciences USA* **109**, 9929–9934 (2012).

[35] van Segbroeck, S., Pacheco, J. M., Lenaerts, T. & Santos, F. C. Emergence of fairness in repeated group interactions. *Physical Review Letters* **108**, 158104 (2012).

[36] Stewart, A. J. & Plotkin, J. B. From extortion to generosity, evolution in the iterated prisoner's dilemma. *Proceedings of the National Academy of Sciences USA* **110**, 15348–15353 (2013).

[37] Stewart, A. J. & Plotkin, J. B. Collapse of cooperation in evolving games. *Proceedings of the National Academy of Sciences USA* **111**, 17558 – 17563 (2014).

[38] Knight, V., Harper, M., Glynatsi, N. & Campbell, O. Evolution reinforces cooperation with the

emergence of self-recognition mechanisms: An empirical study of strategies in the Moran process for the iterated prisoner's dilemma. *PLoS One* **13**, e0204981 (2018).

[39] Park, P. S., Nowak, M. A. & Hilbe, C. Cooperation in alternating interactions with memory constraints. *Nature Communications* **13**, 737 (2022).

[40] Chen, X. & Fu, F. Outlearning extortioners: Unbending strategies can foster reciprocal fairness and cooperation. *PNAS Nexus* **2**, pgad176 (2023).

[41] Hilbe, C., Šimsa, S., Chatterjee, K. & Nowak, M. A. Evolution of cooperation in stochastic games. *Nature* **559**, 246–249 (2018).

[42] Barfuss, W., Donges, J. F. & Kurths, J. Deterministic limit of temporal difference reinforcement learning for stochastic games. *Physical Review E* **99**, 043305 (2019).

[43] Kleshnina, M., Hilbe, C., Šimsa, S., Chatterjee, K. & Nowak, M. The effect of environmental information on evolution of cooperation in stochastic games. *Nature Communications* **14**, 4153 (2023).

[44] Bernheim, D. & Whinston, M. D. Multimarket contact and collusive behavior. *The RAND Journal of Economics* **21**, 1–26 (1990).

[45] Matsushima, H. Multimarket contact, imperfect monitoring, and implicit collusion. *Journal of Economic Theory* **98**, 158–178 (2001).

[46] Reiter, J. G., Hilbe, C., Rand, D. G., Chatterjee, K. & Nowak, M. A. Crosstalk in concurrent repeated games impedes direct reciprocity and requires stronger levels of forgiveness. *Nature Communications* **9**, 1–8 (2018).

[47] Donahue, K., Hauser, O. P., Nowak, M. A. & Hilbe, C. Evolving cooperation in multichannel games. *Nature Communications* **11**, 1–9 (2020).

[48] Dal Bó, P. & Fréchette, G. R. On the determinants of cooperation in infinitely repeated games: A survey. *Journal of Economic Literature* **56**, 60–114 (2018).

[49] Traulsen, A., Semmann, D., Sommerfeld, R. D., Krambeck, H.-J. & Milinski, M. Human strategy updating in evolutionary games. *Proceedings of the National Academy of Sciences USA* **107**, 2962–2966 (2010).

[50] Grujic, J., Fosco, C., Araujo, L., Cuesta, J. & Sanchez, A. Social experiments in the mesoscale: Humans playing a spatial prisoner's dilemma. *PLoS One* **5**, e13749 (2010).

[51] Gracia-Lázaro, C. *et al.* Heterogeneous networks do not promote cooperation when humans play a prisoner's dilemma. *Proceedings of the National Academy of Sciences USA* **109**, 12922–12926 (2012).

[52] Grujic, J. *et al.* A comparative analysis of spatial prisoner's dilemma experiments: Conditional cooperation and payoff irrelevance. *Scientific Reports* **4**, 4615 (2014).

[53] Fischbacher, U. & Gächter, S. Social preferences, beliefs, and the dynamics of free riding in public goods experiments. *American Economic Review* **100**, 541–556 (2010).

[54] Martinez-Martinez, I. & Normann, H. Cooperation in multiplayer dilemmas. *SSRN* http://dx.doi.org/10.2139/ssrn.4294660 (2022).

[55] Grujić, J., Eke, B., Cabrales, A., Cuesta, J. A. & Sánchez, A. Three is a crowd in iterated prisoner's

dilemmas: experimental evidence on reciprocal behavior. *Scientific reports* **2**, 1–7 (2012).

[56] Fehl, K., van der Post, D. J. & Semmann, D. Co-evolution of behaviour and social network structure promotes human cooperation. *Ecology letters* **14**, 546–551 (2011).

[57] Rand, D. G., Arbesman, S. & Christakis, N. A. Dynamic social networks promote cooperation in experiments with humans. *Proceedings of the National Academy of Sciences USA* **108**, 19193–19198 (2011).

[58] Bednarik, P., Fehl, K. & Semmann, D. Costs for switching partners reduce network dynamics but not cooperative behaviour. *Proceedings of the Royal Society B: Biological Sciences* **281**, 20141661 (2014).

[59] Cason, T. N., Savikhin, A. C. & Sheremeta, R. M. Behavioral spillovers in coordination games. *European Economic Review* **56**, 233–245 (2012).

[60] Savikhin, A. C. & Sheremeta, R. M. Simultaneous decision-making in competitive and cooperative environments. *Economic Inquiry* **51**, 1311–1323 (2013).

[61] Bednar, J., Chen, Y., Liu, T. X. & Page, S. Behavioral spillovers and cognitive load in multiple games: An experimental study. *Games and Economic Behavior* **74**, 12–31 (2012).

[62] Liu, T. X., Bednar, J., Chen, Y. & Page, S. Directional behavioral spillover and cognitive load effects in multiple repeated games. *Experimental Economics* **22**, 705–734 (2019).

[63] Yang, J., Kawamura, T. & Ogawa, K. Experimental multimarket contact inhibits cooperation. *Metroeconomica* **67**, 21–43 (2016).

[64] Falk, A., Fischbacher, U. & Gächter, S. Living in two neighborhoods? Social interaction effects in the laboratory. *Economic Inquiry* **51**, 563–578 (2013).

[65] McCarter, M. W., Samek, A. & Sheremeta, R. M. Divided loyalists or conditional cooperators? creating consensus about cooperation in multiple simultaneous social dilemmas. *Group & Organization Management* **39**, 744–771 (2014).

[66] Krieg, J. & Samek, A. When charities compete: A laboratory experiment with simultaneous public goods. *Journal of behavioral and experimental economics* **66**, 40–57 (2017).

[67] Angelovski, A., Di Cagno, D., Güth, W., Marazzi, F. & Panaccione, L. Behavioral spillovers in local public good provision: an experimental study. *Journal of Economic Psychology* **67**, 116–134 (2018).

[68] Laferriere, V., Montez, J., Roux, C. & Thöni, C. Multigame contact: A double-edged sword for cooperation. *AEJ: Microeconomics* (forthcoming) (2023).

[69] Nowak, M. A. & Sigmund, K. Tit for tat in heterogeneous populations. *Nature* **355**, 250–253 (1992).

[70] Baek, S. K., Jeong, H. C., Hilbe, C. & Nowak, M. A. Comparing reactive and memory-one strategies of direct reciprocity. *Scientific Reports* **6**, 25676 (2016).

[71] Selten, R. Reexamination of the perfectness concept for equilibrium points in extensive games. *International Journal of Game Theory* **4**, 25–55 (1975).

[72] Stevens, J. R., Volstorf, J., Schooler, L. J. & Rieskamp, J. Forgetting constrains the emergence of cooperative decision strategies. *Frontiers in Psychology* **1**, 235 (2011).

[73] Volstorf, J., Rieskamp, J. & Stevens, J. R. The good, the bad, and the rare: Memory for partners in

social interactions. *PloS one* **6**, e18945 (2011).

[74] Dolan, P. & Galizzi, M. M. Like ripples on a pond: Behavioral spillovers and their implications for research and policy. *Journal of Economic Psychology* **47**, 1–16 (2015).

[75] Maki, A. *et al.* Meta-analysis of pro-environmental behaviour spillover. *Nature Sustainability* **2**, 307–315 (2019).

[76] Rabin, M. & Weizsäcker, G. Narrow bracketing and dominated choices. *American Economic Review* **99**, 1508–1543 (2009).

[77] Chen, D. L., Schonger, M. & Wickens, C. oTree – An open-source platform for laboratory, online, and field experiments. *Journal of Behavioral and Experimental Finance* **9**, 88–97 (2016).

[78] Ichinose, G. & Masuda, N. Zero-determinant strategies in finitely repeated games. *Journal of Theoretical Biology* **438**, 61–77 (2018).
