# Supplementary material for: Dynamics of cooperation in concurrent games
Source: Nat Commun. 2025 Feb 11;16:1524. doi: 10.1038/s41467-025-56083-7 (PMC11814277; doi:10.1038/s41467-025-56083-7)
Supplement: Supplementary file 2 — Reporting Summary [file 41467_2025_56083_MOESM2_ESM.pdf]

nature portfolio

Corresponding author(s): Charlotte Rossetti

Last updated by author(s): Dec 9, 2024

Reporting Summary

Nature Portfolio wishes to improve the reproducibility of the work that we publish. This form provides structure for consistency and transparency in reporting. For further information on Nature Portfolio policies, see our Editorial Policies and the Editorial Policy Checklist.

Statistics

For all statistical analyses, confirm that the following items are present in the figure legend, table legend, main text, or Methods section.

n/a	Confirmed	
☐	☒	The exact sample size (*n*) for each experimental group/condition, given as a discrete number and unit of measurement
☐	☒	A statement on whether measurements were taken from distinct samples or whether the same sample was measured repeatedly
☐	☒	The statistical test(s) used AND whether they are one- or two-sided<br>*Only common tests should be described solely by name; describe more complex techniques in the Methods section.*
☒	☐	A description of all covariates tested
☒	☐	A description of any assumptions or corrections, such as tests of normality and adjustment for multiple comparisons
☐	☒	A full description of the statistical parameters including central tendency (e.g. means) or other basic estimates (e.g. regression coefficient) AND variation (e.g. standard deviation) or associated estimates of uncertainty (e.g. confidence intervals)
☐	☒	For null hypothesis testing, the test statistic (e.g. *F*, *t*, *r*) with confidence intervals, effect sizes, degrees of freedom and *P* value noted<br>*Give P values as exact values whenever suitable.*
☒	☐	For Bayesian analysis, information on the choice of priors and Markov chain Monte Carlo settings
☒	☐	For hierarchical and complex designs, identification of the appropriate level for tests and full reporting of outcomes
☐	☒	Estimates of effect sizes (e.g. Cohen's *d*, Pearson's *r*), indicating how they were calculated

Our web collection on statistics for biologists contains articles on many of the points above.

Software and code

Policy information about availability of computer code

Data collection	All evolutionary simulations were performed using Matlab R2020b.<br>The online experiment was implemented using oTree (Version: 3.4.0), an open-source platform for web-based interactive tasks run in Python (Version: 3.8.3).
Data analysis	To analyze the experimental data, we used R (Version: 4.0.4)

For manuscripts utilizing custom algorithms or software that are central to the research but not yet described in published literature, software must be made available to editors and reviewers. We strongly encourage code deposition in a community repository (e.g. GitHub). See the Nature Portfolio guidelines for submitting code & software for further information.

Data

Policy information about availability of data

All manuscripts must include a data availability statement. This statement should provide the following information, where applicable:

- Accession codes, unique identifiers, or web links for publicly available datasets
- A description of any restrictions on data availability
- For clinical datasets or third party data, please ensure that the statement adheres to our policy

The data is publicly available at: http://OSF.IO/XQGHF

Research involving human participants, their data, or biological material

Policy information about studies with human participants or human data. See also policy information about sex, gender (identity/presentation), and sexual orientation and race, ethnicity and racism.

Reporting on sex and gender	Gender was recorded through self-reporting but not used in the analysis.
Reporting on race, ethnicity, or other socially relevant groupings	We do not report ethnicity or social groups.
Population characteristics	See above
Recruitment	Participants were recruited through the online platform Prolific. The sample was restricted to UK residents.
Ethics oversight	Ethics Committee of the Medical Faculty of Kiel University (D 571/20)

Note that full information on the approval of the study protocol must also be provided in the manuscript.

Field-specific reporting

Please select the one below that is the best fit for your research. If you are not sure, read the appropriate sections before making your selection.

☐ Life sciences ☒ Behavioural & social sciences ☐ Ecological, evolutionary & environmental sciences

For a reference copy of the document with all sections, see nature.com/documents/nr-reporting-summary-flat.pdf

Behavioural & social sciences study design

All studies must disclose on these points even when the disclosure is negative.

Study description	The study is a quantitative experiment ran online.
Research sample	We recruited N = 316 participants through the University of Exeter student pool - FEELE (Finance and Economics Experimental Laboratory at Exeter). The sample was made of 161 females (excluding the 4 participants who dropped out.)
Sampling strategy	The sample is a convenience sample. Sampling size was based on previous similar studies and reflected the total number of participants we would hope to achieve from the pool we chose.
Data collection	The economic experiments data was collected via the platform oTree. The interactive task is run fully online without the presence of experimenter. Once the experiment is programmed and launched on a recruitment platform (University of Exeter student pool - FEELE), data collection proceeds automatically and independently of the researcher who is only available for questions via emails. Once the data collection is completed, the data can be downloaded as a csv file for offline analysis.
Timing	Data was collected between 29.04.21-19.05.21 and again between 24.09.21 and 22.10.21 over 15 sessions. Each session recruited up to 32 participants and would stop once all participants completed the task.
Data exclusions	No data was excluded for the analysis
Non-participation	Four participants dropped out without giving any reason after giving their consent to participate.
Randomization	Treatments were pseudo-randomized across session to have a spread of day of the weeks and times. Participants signed up for the session date and time of their choice without knowledge of the treatment.

Reporting for specific materials, systems and methods

We require information from authors about some types of materials, experimental systems and methods used in many studies. Here, indicate whether each material, system or method listed is relevant to your study. If you are not sure if a list item applies to your research, read the appropriate section before selecting a response.

Materials & experimental systems

n/a	Involved in the study
☒	☐ Antibodies
☒	☐ Eukaryotic cell lines
☒	☐ Palaeontology and archaeology
☒	☐ Animals and other organisms
☒	☐ Clinical data
☒	☐ Dual use research of concern
☒	☐ Plants

Methods

n/a	Involved in the study
☒	☐ ChIP-seq
☒	☐ Flow cytometry
☒	☐ MRI-based neuroimaging

Plants

Seed stocks

Report on the source of all seed stocks or other plant material used. If applicable, state the seed stock centre and catalogue number. If plant specimens were collected from the field, describe the collection location, date and sampling procedures.

Novel plant genotypes

Describe the methods by which all novel plant genotypes were produced. This includes those generated by transgenic approaches, gene editing, chemical/radiation-based mutagenesis and hybridization. For transgenic lines, describe the transformation method, the number of independent lines analyzed and the generation upon which experiments were performed. For gene-edited lines, describe the editor used, the endogenous sequence targeted for editing, the targeting guide RNA sequence (if applicable) and how the editor was applied.

Authentication

Describe any authentication procedures for each seed stock used or novel genotype generated. Describe any experiments used to assess the effect of a mutation and, where applicable, how potential secondary effects (e.g. second site T-DNA insertions, mosiacism, off-target gene editing) were examined.
